# Case report: Neonatal-onset inflammatory bowel disease due to novel compound heterozygous mutations in *DUOX2*

**DOI:** 10.3389/fgene.2023.1276697

**Published:** 2023-11-23

**Authors:** Andrea Finocchi, Lucia Pacillo, Maria Chiriaco, Gigliola Di Matteo, Paola Francalanci, Giulia Angelino, Tamara Caldaro, Beatrice Rivalta, Maurice O’Mara, Suisheng Zhang, Francesca Romana Lepri, Antonio Novelli, Paola De Angelis, Ulla G. Knaus, Francesca Rea

**Affiliations:** ^1^ Research Unit of Primary Immunodeficiencies, Unit of Clinical Immunology and Vaccinology, IRCCS Bambino Gesù Children Hospital, Rome, Italy; ^2^ Department of Systems Medicine, University of Rome Tor Vergata, Rome, Italy; ^3^ IRCCS Bambino Gesù Children’s Hospital, Unit of Pathology, Rome, Italy; ^4^ IRCCS Bambino Gesù Children’s Hospital, Digestive Endoscopy and Surgery Unit, Rome, Italy; ^5^ School of Medicine, Conway Institute, University College Dublin, Dublin, Ireland; ^6^ Laboratory of Medical Genetics, IRCCS Bambino Gesù Children’s Hospital, Translational Cytogenomics Research Unit, Rome, Italy

**Keywords:** inflammatory bowel disease, neonatal-IBD, NADPH oxidase, DUOX2, VEO-IBD

## Abstract

Very Early Onset Inflammatory Bowel Disease (VEO-IBD) is potentially associated with genetic disorders of the intestinal epithelial barrier or inborn errors of immunity (IEI). Dual oxidase 2 (DUOX2), an H_2_O_2_-producing NADPH oxidase expressed at apical enterocyte membranes, plays a crucial role in innate defense response. Biallelic *DUOX2* mutations have been described only in two patients with VEO-IBD to date. We report the case of a 1-month-old female infant who presented persistent high C-reactive protein (CRP) levels from birth and anemia. Positive occult blood and very high calprotectin in the stool were detected and abdominal ultrasound showed thickened last ileal loop. Full endoscopy evaluation revealed important colon stenosis with multiple pseudo-polyploidy formations that resulted refractory to steroid therapy, requiring a partial colic resection. Histological examination of biopsy samples showed morphological features of IBD. Whole Exome Sequencing (WES) disclosed compound heterozygous variants in the *DUOX2* gene: the pathogenic c.2524C>T; p.Arg842Ter and the variant of uncertain significance (VUS) c.3175C>T; p.Arg1059Cys. Molecular and functional studies showed the presence of mutant DUOX2 in the intestinal epithelium of the patient, albeit with at least 50% decreased catalytic activity. In conclusion, we describe the third patient to date with compound heterozygous variants of *DUOX2*, responsible for monogenic neonatal-IBD. This case expands the knowledge about Mendelian causes of VEO-IBD and DUOX2 deficiency. We suggest that DUOX2 should be part of the diagnostic evaluation of patients with suspected monogenic VEO-IBD.

## 1 Introduction

Inflammatory bowel disease (IBD) is a multifactorial disorder caused by dysregulated immune responses to commensal or pathogenic intestinal microbes resulting in chronic intestinal inflammation. The interplay between innate and adaptive immunity, along with factors such as host genetic susceptibility and intestinal microbiota, influences the severity of symptoms and the age at onset. The genetics of IBD are complex, with IBD conventionally defined as polygenic disorders that include a spectrum of rare diseases, which manifest with IBD-like intestinal inflammation. Clinically, age at diagnosis is important, and it appears that very-early-onset IBD (VEO-IBD, age <6 years at diagnosis), infantile (toddler, age <2 years at diagnosis), as well as neonatal onset (age <28 days at diagnosis) might be distinct forms of the disease, which are phenotypically and genetically different from older-onset IBD (Kammermeier et al., 2023).

In the last decade, significant progress has been made in our understanding of how genetics play a role in the pathogenesis of IBD. To date, over 230 risk loci have been identified by next-generation sequencing (NGS), including 90 genes responsible for monogenic IBD. In these patients, IBD is caused by high penetrance genetic variants in a single gene (monogenic IBD). The relative contribution of genetic factors and the frequency of IBD causing monogenic variants seem to be inversely related to the age of onset of IBD (Collen et al., 2022). Several studies identified the lack of reactive oxygen species (ROS) production by NADPH oxidases expressed in phagocytes (NOX2) and in intestinal epithelial cells (NOX1 and DUOX2) as risk factors for VEO- or adult-IBD. The intestinal dual oxidase DUOX2 is a heterodimeric enzyme (DUOX2/DUOX2A) expressed on the apical side of enterocyte membranes that mediates the host defense against microbial infections by producing hydrogen peroxide (H_2_O_2_), thereby sustaining intestinal immune homeostasis (Hayes et al., 2015).

Here, we describe the identification and characterization of novel compound heterozygous variants in *DUOX2* by Whole Exome Sequencing (WES) in a patient diagnosed with neonatal-onset IBD, showing a somewhat different phenotype, and thus expanding the genotypic and phenotypic spectrum of DUOX2 deficiency.

## 2 Methods

### 2.1 Patient and informed consent

All procedures performed in the study were in accordance with the ethical standards of the institutional research committee and with the 1964 Declaration of Helsinki and its later amendments. Informed consent, following standard ethical procedures with approval of the Children’s Hospital Bambino Gesù Ethical Committee, was obtained from the case-index patient and her parents.

### 2.2 Trio-based WES analysis

Whole exome sequencing was performed on the proband’s and parents’ genomic DNA, extracted from peripheral blood, using a Twist Human Core Exome Kit (Twist Bioscience), according to the manufacturer’s protocol, and sequenced on an Illumina NovaSeq6000 platform. The reads were aligned to the human genome build GRCh37/UCSC hg19. Based on the guidelines of the ACMG, a minimum depth coverage of 30X was considered suitable for analysis. The BaseSpace pipeline and the Geneyx software LifeMap Sciences were respectively used for variant calling and annotating variants. The variants were filtered by *in silico* analysis of all the genes associated with IBD. The complete list of the genes included in the panel is reported in the Supplementary Data.

### 2.3 Cell culture and transfection

NCI-H661 cells were cultivated in RPMI 1640 medium with 10% fetal bovine serum and 1% sodium pyruvate. DUOX2 variant c.3175C>T (p.Arg1059Cys) was introduced into HA-tagged DUOX2 WT in pcDNA3.1 by site-directed mutagenesis. HA-DUOX2 WT, HA-DUOX2 R1059C, and pcDNA3.1 plasmids (EV) were transfected in H661 cells stably expressing DUOXA2 using FuGene HD (Promega) for 48 h.

### 2.4 Protein isolation and Western Blotting

Cell lysates in RIPA buffer containing protease inhibitors were separated by SDS-PAGE and immunoblotted with HA (Covance Laboratories, Princeton, NJ), DUOX2 (custom, DUOX2 aa775-1026), and actin (Sigma-Aldrich, St. Louis, MO) antibodies, followed by secondary horseradish peroxidase-conjugated anti-rabbit (Southern Biotech) or anti-mouse antibody (Cell Signalling, Danvers, MA). Proteins were visualized using ECL reagent (Pierce, Waltham, MA).

### 2.5 Reactive oxygen species assay

H_2_O_2_ generation by DUOX2, stimulated by the addition of 100 ng/mL PMA and 1 µM thapsigargin for 1 h, was measured using a homovanillic acid assay. H_2_O_2_ production was standardized to H_2_O_2_ standard curves, and the cell lysate protein concentration was measured using a BCA assay.

### 2.6 Microscopy

Tissue sections on slides were deparaffinized, followed by citrate buffer mediated antigen retrieval. After blocking (5% goat serum, 1% BSA in PBS), tissues were incubated with DUOX2 antibody (custom, DUOX2 aa634-648) at 4°C overnight. After washing, tissues were incubated with anti-rabbit Alexa Fluor 647, then 300 mmol/L DAPI, and mounted. Images were acquired using a Zeiss LSM 800 airyscan microscope with a Plan-Apochromat ×63 oil immersion objective.

## 3 Results

### 3.1 Clinical features of the patient

A 1-month-old female infant born at term from non-consanguineous parents presented persistent high C-reactive protein (CRP) levels from birth, without other symptoms or signs of systemic inflammation like fever or rash, and normal serum procalcitonin levels. Blood tests performed on the first day of life for routine monitoring, as her mother’s vaginal swab and uroculture resulted positive for *S. haemolyticus* and *E. coli*, respectively, showed a serum CRP level of 2 mg/dL (reference value <0.05 mg/dL). Despite receiving a 4 weeks course of broad-spectrum antibiotic therapy and negative results from comprehensive microbiological investigations, high CRP persisted. Moreover, the infant developed normochromic and normocytic anemia with 8.5 gr/dl and normal iron deposits.

At the age of 1 month and 11 days, she was admitted to our hospital for further investigations. At admission, her height and weight were within the normal limits. Physical examination was normal, and the perianal region was unremarkable for fistulas or fissures. Hemoglobin was 8 g/dL, MCV was 91.3 fL, white blood cell count was 10.840/mm^3^, thrombocyte count was 796.000/mm^3^, CRP was 2.1 mg/dL (normal level <00.5 mg/dL), and procalcitonin was 0.09 ng/mL (normal level <0.5 ng/mL). Occult blood testing was positive and a very high fecal calprotectin level of 2,812 µg/gr (reference value negative <30 µg/gr) was detected in the stool. Cow’s milk–induced colitis was suspected, but the infant did not respond to elimination diet. An extended immunological work up with serum immunoglobulins level, multiparameter flow cytometry analysis of peripheral blood leucocytes, including naïve and memory CD4^+^ and CD8+T cells, recent thymic emigrant cells, as well as B cell subsets resulted normal. Respiratory burst activity by dihydrorhodamine (DHR) as well as NBT-test were normal. Specific IgE for cow’s milk were negative. Anti-*Saccharomyces cerevisiae* antibody (ASCA) IgG, anti-neutrophil cytoplasmic antibody (ANCA), and ferritin levels were in the normal range.

**FIGURE 1:**
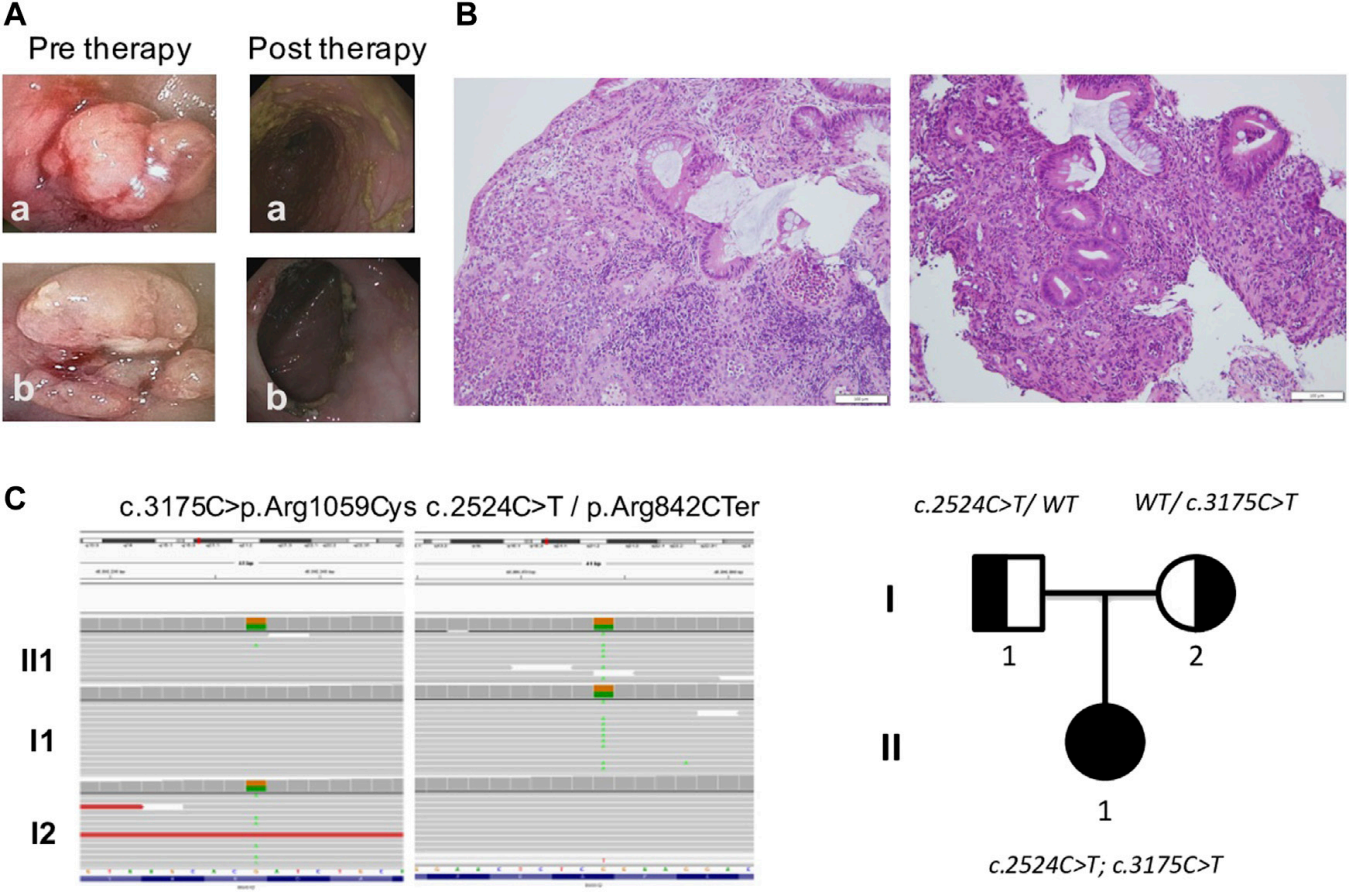
Gut investigations and molecular characterization. **(A)** Endoscopic images revealed two polyploid lesions of the left colon with underlying stricture of lumen which prevented further progression of the endoscope. **(B)** Histology showed chronic active colitis with rarefaction and distortion of the glandular crypts and heavy diffuse inflammatory infiltrate, including lymphocytes (CD4+++, CD8−), plasma cells, neutrophils, and eosinophils in the lamina propria with crypt abscesses. Goblet cell depletion; no granulomas or giant cell reaction were present. **(C)** WES results featuring mutations found in DUOX2 and genealogical tree showing hereditary pattern of disease.

Abdominal ultrasound revealed thickening of the transverse colon wall and of the last ileal loop. Endoscopic examination showed two polyploid lesions on the left colon, with underlying stricture of the lumen that prevented further progression of the endoscope. Left colonic sigma and rectum mucosa was fragile, with multiple erosions of mild intensity (Figure 1A). In contrast, esophagogastroduodenoscopy showed a macroscopically normal esophagus and duodenum, with no perianal or extra-intestinal manifestations. Pathology was remarkable for moderate chronic active aspecific colitis at the left colon, sigmoid colon, and rectum. Water-soluble contrast enema was performed to study colonic transit, which confirmed the obstruction at the splenic flexure of the colon with further progression of contrast.

### 3.2 Patient management

The patient was started on methylprednisolone IV 1 mg/kg/day for 15 days, with normalization of CRP and Hb. The patient underwent a second endoscopy but polyploid lesions and stenosis were refractory to medical therapy, requiring a partial colon resection. Histological examination of surgical colonic samples showed chronic active colitis, with rarefaction and distortion of the glandular crypts and heavy diffuse inflammatory infiltrate, including lymphocytes (CD4+++, CD8^−^), plasma cells, neutrophils, and eosinophils in the lamina propria with crypt abscesses (Figure 1B). Goblet cell depletion was apparent, while granulomas or giant cell reaction were not present. A preliminary diagnosis of VEO-IBD colitis phenotype with stenosis was made. After surgery, the patient was treated with metronidazole and subsequently started on mesalamine 40 mg/kg once a day as maintenance therapy.

### 3.3 WES and variants classification

Considering the neonatal onset of IBD, WES analysis was performed on the index-case patient and her parents. Two different variants in *DUOX2* (NM_014080) were found: the c.2524C>T mutation inherited from her father and the c.3175C>T variant inherited from her mother (Figure 1C). The c.2524C>T mutation introduced a nonsense variant p.Arg842Ter in DUOX2 and is considered pathogenic. This variant, previously identified in patients with congenital hypothyroidism (Fu et al., 2016), is described in population databases (rs119472028, gnomAD 0.008%) and ClinVar (Variation ID: 4064). The second variant c.3175C>T induced a missense p.Arg1059Cys in DUOX2 protein that is considered of uncertain significance. This variant is present in population databases (rs145502900, gnomAD 0.04%) and ClinVar (Variation ID: 1520364).

Moreover, two variants of uncertain significance (VUS) have been identified in other potentially relevant genes and are reported in the Supplementary Data.

### 3.4 Functional analysis

**FIGURE 2:**
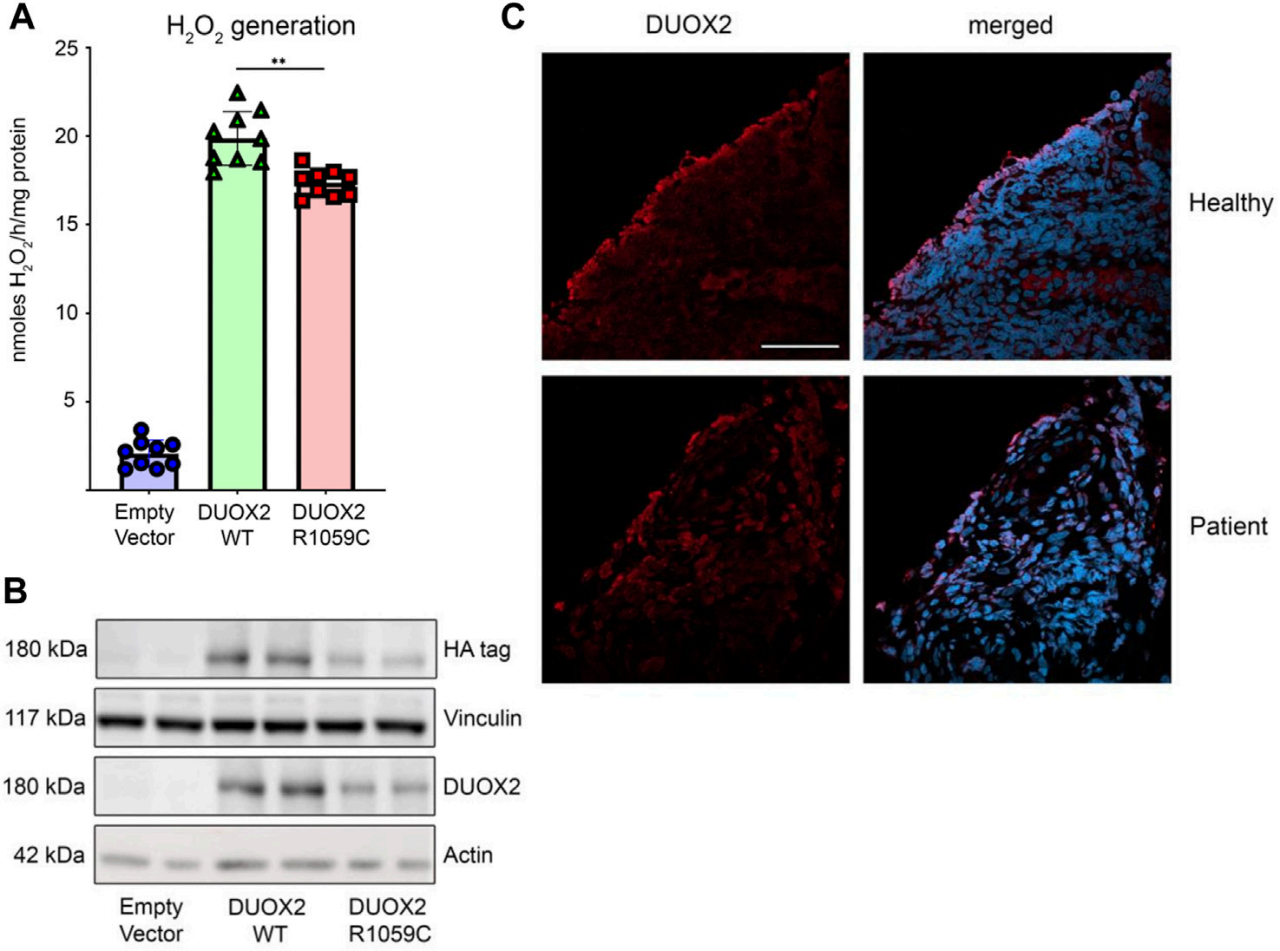
Functional characterization of DUOX2 mutation R1059C. **(A)** H202 generation by DUOX2 WT and DUOX2 R1059C in H661-DUOXA2 cells; stimulation with PMA (10Ong/m1) and thapsigargin (1 kM); empty vector served as negative control. Error bars +/‐ standard deviation (n = 9); Brown-Forsythe and Welch one-way ANOVA with Dunnett's T3 post hoc test **I3 5 0.005. **(B)** Expression of HA-DUOX2 WT and HA-DUOX2 R1059C in H661- DUOXA2 cells; vinculin and actin served as loading controls. **(C)** Immunofluorescence microscopy of DUOX2 (red) stained biopsy sections from a healthy donor and the patient confirming apical membrane localization; DAPI for nuclei (blue) (scale bar 50F.tm).

The functional importance of the DUOX2 mutations was assessed. DUOX2 p.Arg842Ter (DUOX2 R842X) introduces a premature stop codon in one of the two calcium-binding EF-hand regions, leading to a truncated enzyme that lacks the structurally conserved NOX domain (M1-M6) essential for NADPH oxidase catalytic activity (Wu et al., 2021). This nonsense mutation will result in a short, non-functional DUOX2 protein that may be degraded. The missense mutation on the second allele, p.Arg1059Cys (DUOX2 R1059C), replacing hydrophilic arginine with cysteine, is located in the second transmembrane domain M1. DUOX2 R1059 is homologous to R1062 in DUOX1, which was predicted to be part of a hydrophilic tunnel for oxygen substrate entrance and product exit (Wu et al., 2021). To determine the catalytic activity of the DUOX2 R1059C mutation, NCI-H661 cells stably transfected with wildtype DUOXA2, an essential DUOX2 dimerization partner, were used to express either the HA-tagged DUOX2 wildtype or the DUOX2 R1059C mutant. Expression of mutant DUOX2 was altered, and stimulated H_2_O_2_ generation was significantly decreased (10%–15%) when compared to wildtype DUOX2 (Figures 2A,B). Compound heterozygous mutations in DUOX2 can lead to defective hetero-tetramer assembly and loss of crypt cell surface expression (Parlato et al., 2017). Immunofluorescence microscopy of patient tissue samples revealed DUOX2 expression at the apical side of the intestinal epithelium, indicating that DUOX2 expression and translocation was preserved, albeit that available antibodies may also detect truncated DUOX2 if the protein remains stably expressed (Figure 2C).

### 3.5 Diagnosis and follow-up

Molecular and functional studies showed expression of mutant DUOX2 in the intestinal epithelium of the patient, albeit with decreased catalytic activity (at least 50% H_2_O_2_ output reduction) due to a monoallelic truncating variant, leading to the diagnosis of neonatal-onset IBD due to *DUOX2* mutations. The presence of this nonsense c.2524C>T mutation in *DUOX2*, reported in patients with congenital hypothyroidism, led us to investigate the thyroid function of the patient, which was normal. Endoscopic follow-up performed 6 months after surgery showed mucosal healing, with minimal histological chronic activity. Currently, 1 year later, the patient is on mesalamine treatment and is in clinical and biochemical remission, with normal fecal calprotectin levels that are monitored every 3 months.

## 4 Discussion

With the expanding use of next-generation sequencing platforms in patients with IBD phenotypes, the number of monogenic disorders associated with intestinal inflammation is steadily growing. DUOX2 is an enzyme belonging to the NADPH oxidase family, expressed at the apical membrane of enterocytes in all gut segments. Its expression is upregulated by microbiota and inflammation-derived signals. DUOX2 plays a crucial role in innate defense response and intestinal homeostasis by producing hydrogen peroxide (H_2_O_2_). Taking into account the physiological role of DUOX2 in innate immunity and intestinal homeostasis, it is not surprising that variants in *DUOX2* would actively contribute to pathological states in the gastrointestinal tract, such as IBD.

**FIGURE 3:**
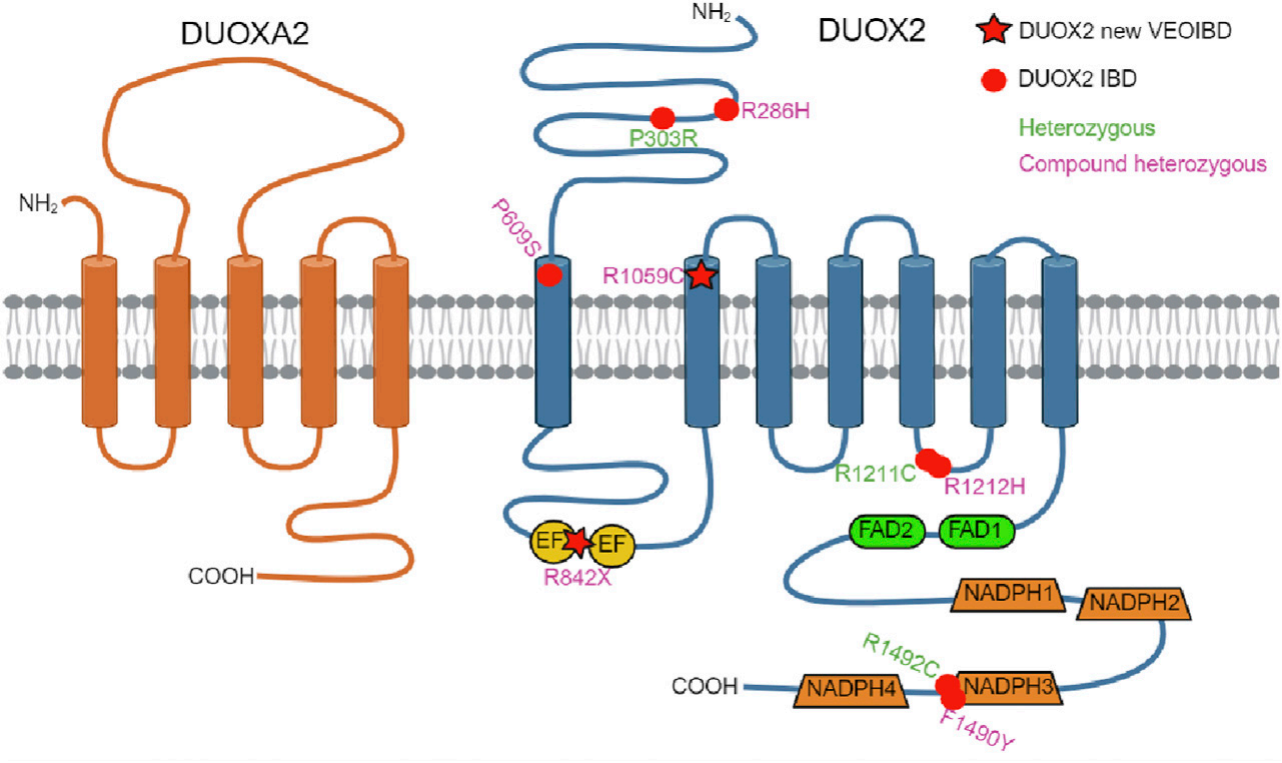
DUOX2/DUOXA2 topology model. Location of IBD-associated DUOX2 variants identified in this study (R842X/R1059C, star symbol) and in earlier studies (dot symbol) including compound heterozygous DUOX2 variants (R286H/P609S, R1212H/F1490Y) and heterozygous DUOX2 variants (P303R, R1211C, R1492C) is shown. EF (EF-hand calcium-binding motif); created with BioRender.com.

Very rare monoallelic LOF missense variants in *DUOX2* have been reported as risk factors for VEO-IBD (Figure 3). (Hayes et al., 2015; O’Neill et al., 2015) Hayes et al. (2015) first identified novel heterozygous *DUOX2* variants in two patients with VEO-IBD, demonstrating reduced ROS production, Paneth cell metaplasia, and defective host resistance to *C. jejuni*. Exome sequencing revealed a heterozygous *DUOX2* missense variant shared by 15 members of the same Ashkenazi Jewish family with a high incidence of adult-onset Crohn Disease (CD) (Levine et al., 2016). Biallelic *DUOX2* mutations have been described in only two patients with VEO-IBD to date (Parlato et al., 2017; Kyodo et al., 2022). Parlato et al. (2017) first identified novel biallelic *DUOX2* variants in a 3-year-old Italian male with pancolitis, showing impaired H_2_O_2_ generation and loss of DUOX2 expression in the intestinal tissue of the patient. Kyodo et al. (2022) recently reported the second case of VEO-IBD associated with biallelic *DUOX2* variants leading to a 50% reduction of H_2_O_2_ generation by each of the missense mutations. The patient had pancolitis and extensive small intestinal lesions, requiring various treatments, and long-term clinical remission was achieved with ustekinumab.

Here, we describe a novel compound heterozygous mutation in *DUOX2* that leads to protein expression but at least 50% decreased H_2_O_2_ generation. The patient described here exhibits a somewhat different phenotype compared to previously reported cases. Our case is the youngest case and the first with neonatal-onset IBD associated with inactivating *DUOX2* mutations in the literature. Neonatal or infantile-onset IBD accounts for only 1% of pediatric cases, and is often associated with primary immune deficiency disorders, especially those caused by mutations in *IL-10*, *XIAP*, *NCF2*, *IPEX,* and *TTC7A* (Kelsen et al., 2019). The phenotypes and genotypes of all reported patients with IBD showing *DUOX2* mutations are summarized in Table 1.

**TABLE 1:** IBD patients with DUOX2 variants reported to date. Modified from Kyodo et al. (2022).

Genotype	Sex	Diagnosis	Age at diagnosis	Clinical phenotype	Thyroid function	Therapy	References
R842X/R1059C (compound heterozygous)	F	IBD-U	1 month	Polypoid lesions with lumen stricture	Normal	5-ASA, partial colon resection	Present case report
R1211C (heterozygous)	M	IBD-U	4 years	Severe pancolitis, perforation	Normal	Colectomy	Hayes et al. (2015)
R1492C (heterozygous)	M	UC	4 years	Pancolitis	Normal	NR	Hayes et al. (2015)
P303R (heterozygous)	NA	CD	Adult (NA)	15 members of the same Ashkenazi Jewish family with adult-onset CD	Normal	Variable	Levine et al. (2016)
R286H/P609S (compound heterozygous)	M	IBD	3 years	Pancolitis	Normal	AZA intolerance, corticosteroid dependence, 5-ASA	Parlato et al. (2017)
R1212H/F1490Y (compound heterozygous)	M	CD	1 year	Pancolitis	Normal	5-ASA and AZA intolerance, corticosteroid dependence, IFX second failure, remission with UST	Kyodo et al. (2022)

M, male; F, female; IBD, inflammatory bowel disease; IBD-U, inflammatory bowel disease unclassified; UC, ulcerative colitis; CD, Crohn’s disease; 5-ASA, mesalazine; AZA, azathioprine; IFX, infliximab; UST, ustekinumab; NA, not available.

Mutations in *DUOX2* represent the most common cause of congenital hypothyroidism, secondary to the loss of H_2_O_2_ production in thyroid follicular cells (O’Neill et al., 2015; Kizys et al., 2017). Currently, the thyroid function in our patient and in all previously reported patients with VEO-IBD due to *DUOX2* mutations was normal. Of note, Grasberger et al. (2018) reported that patients with congenital hypothyroidism could be at an increased risk of developing IBD. Additionally, *DUOX2* variants with decreased catalytic activity and/or cell surface expression were identified in an adult population without clinically diagnosed hypothyroidism or IBD (Grasberger et al., 2021). The study showed an association of DUOX2 alterations with IL17C upregulation and asymptomatic microbiota dysbiosis, again strengthening the notion that proper functioning of DUOX2 is essential for microbiota-immune homeostasis and intestinal host defense that may precede the manifestation of IBD in adults or will result in VEO-IBD. Exposure to pathogens or pathobionts strongly induces epithelial DUOX2 expression as a host defense mechanism, and it is under those circumstances that DUOX2 haploinsufficiency may provide the critical input for triggering DUOX2 LOF variant-associated VEO-IBD. The thus far seemingly mutually exclusive manifestation of VEO-IBD *versus* hypothyroidism in patients with DUOX2 inactivating variants may be connected to diverging tissue and signaling pathway specific contexts and allele penetrance, an area that requires more in-depth investigation.

The gastrointestinal involvement in our patient was unusual, characterized by two polyps of the left colon, with underlying stricture of the lumen that prevented further progression during endoscopy. Patients with monogenic IBD are more likely to exhibit a Crohn’s disease phenotype with higher rates of stricturing, penetrating disease, and extra-intestinal manifestation, compared with non-monogenic VEO-IBD (Collen et al., 2022). Perianal disease is common in the monogenic group.

From a clinical point of view, the infant was asymptomatic with no intestinal symptoms such as abdominal pain, rectal bleeding, perianal disease, diarrhea, or intestinal obstruction. The only sign of intestinal involvement was increased inflammatory markers (CRP and calprotectin) and anemia. Of note, she was on an exclusively liquid diet (milk). Additionally, she is currently in clinical remission (1-year post-surgery), solely on mesalamine maintenance therapy. It is well known that infantile-onset is a risk factor for monogenic diseases and that monogenic disease, as opposed to age at diagnosis, is the most important driver of disease severity and bad outcome in VEO-IBD (Collen et al., 2022). Published cases of DUOX2 variants disseminated intestinal inflammation, rapid disease progression, and higher rates of treatment resistance, including azathioprine intolerance and corticosteroid dependence. Nevertheless, due to her young age and short duration of follow-up, it is possible that additional manifestations may develop later in life. The reasons for the different clinical phenotype observed in our case remain unclear. Because of its recent discovery and extreme rarity, the exact role of DUOX2 impairment and the full spectrum of the disease remain undefined. Therefore, it is uncertain whether differences in the disease phenotype could be attributed to distinct mutations or to variable expressivity of the disease, through other genetic and/or environmental factors such as diet, gut microbiota, or infectious insult.

In conclusion, we describe the third patient to date with compound heterozygous variants of DUOX2. This case expands the knowledge about DUOX2 deficiency by describing a patient with neonatal onset of IBD presenting with colonic polyps. We suggest that DUOX2 should be part of the diagnostic evaluation of patients with suspected monogenic IBD. Identification of gene defects causing intestinal inflammation will contribute to a better understanding of intestinal physiology and homeostasis and is a key to guiding specific therapy. Novel therapeutic options, targeting DUOX2-derived ROS signaling, such as ROS inducers, could be considered. Murine data suggest that H_2_O_2_ produced by probiotics, such as *Lactobacillus* strains, promotes epithelial restitution during colitis (Singh et al., 2018). Additional studies are needed to determine whether administration of ROS inducers, such as probiotics, potentially in gut-specific formulations, can alleviate the intestinal manifestations in these patients.

## Data Availability

The data presented in the study are deposited in the dbSNP repository, accession number rs119472028 (c.2524C>T p.Arg842Ter) and rs145502900 (c.3175C>T p.Arg1059Cys).
