# Supplementary material for: Case report: Neonatal-onset inflammatory bowel disease due to novel compound heterozygous mutations in DUOX2
Source: Front Genet. 2023 Nov 23;14:1276697. doi: 10.3389/fgene.2023.1276697 (PMC10701422; doi:10.3389/fgene.2023.1276697)
Supplement: Supplementary file 1 [file DataSheet1.pdf]

Supplementary Data.

List of VUS identified

Gene	Ref.seq	Variant	dbSNP	Genotype	inherited	ClinVar ID
IL6R	NM_000565.4	c.574A>G / p.Met192Val	rs139710914	het	mother	2045111
RTEL1	NM_016434.4	c.217G>T / p.Ala73Ser	rs774311444	het	mother	844435

Gene list of the panel:

ADA,ADA2,ADAM17,AICDA,ATG16L1,BTK,CARD9,CASP8,CD3D,CD3E,CD3G,CD40LG,CDH1,COL7A1,CTLA4,CUL2,CYBA,CYBB,DCLRE1C,DKC1,DOCK8,DUOX2,ENTPD1,EPCAM,FERMT1,FOXP3,G6PC3,GUCY2C,HPS1,HPS4,HPS6,ICOS,IFNG,IKBKG,IL10,IL10RA,IL10RB,IL12B,IL17A,IL18RAP,IL21,IL23R,IL2RA,IL2RG,IRGM,ITGB2,LACC1,LIG4,LRBA,LRRK2,MASP2,MEFV,MVK,NCF1,NCF2,NCF4,NFAT5,NOD2,NOX1,NPC1,PIK3R1,PLA2G4A,PLCG2,PTEN,RAG2,RET,RIPK1,RIPK2,RTEL1,SH2D1A,SKIV2L,SLC26A3,SLC37A4,SLC5A1,SLC9A3,SLCO2A1,STAT1,STAT3,STAT5B,STXBP2,TAOK2,TGFB1,TRIM22,TTC37,TTC7A,WAS,XIAP,ZAP70,ACD,ACP5,ACTB,ADAR,AIRE,AK2,AP1S3,AP3B1,AP3D1,APOL1,ARHGEF1,ARPC1B,ARPC5,ATM,ATP6AP1,B2M,BACH2,BCL10,BCL11B,BLM,BLNK,BLOC1S3,BLOC1S6,BRCA1,BRCA2,BRIP1,CYBC1,C1QA,C1QB,C1QC,C1R,C1S,C2,C3,C4A,C4B,C5,C6,C7,C8A,C8B,C8G,C9,CARD11,CARD14,CARMIL2,CASP10,CCBE1,CD19,CD247,CD27,CD40,CD46,CD55,CD59,CD70,CD79A,CD79B,CD81,CD8A,CDCA7,CEBPE,CFB,CFD,CFH,CFHR1,CFHR2,CFHR3,CFHR4,CFHR5,CFI,CFP,CFTR,CHD7,CIB1,CIITA,CLCN7,CLEC7A,COLEC11,COPA,CORO1A,CR2,CSF2RA,CSF2RB,CTC1,CTPS1,CTSC,CXCR4,DBR1,DCLRE1B,DDX58,DEF6,DGAT1,DNAJC21,DNASE1L3,DNASE2,DNASE2B,DNMT3B,DOCK2,DTNBP1,EFL1,ELANE,EPG5,ERBIN,ERCC4,ERCC6L2,EXTL3,FAAP24,FADD,FANCA,FANCB,FANCC,FANCD2,FANCE,FANCF,FANCG,FANCI,FANCL,FANCM,FAS,FASLG,FAT4,FCGR3A,FCHO1,FCN3,FERMT3,FLT3LG,FOXN1,FPR1,G6PD,GATA2,GFI1,GINS1,HAVCR2,HAX1,HELLS,HMOX1,HPS3,HPS5,HYOU1,ICOSLG,IFIH1,IFNAR1,IFNAR2,IFNGR1,IFNGR2,IGLL1,IKBKB,IKZF1,IKZF3,IL12RB1,IL12RB2,IL17F,IL17RA,IL17RC,IL18BP,IL1RN,IL21R,IL2RB,IL36RN,IL6R,IL6ST,IL7R,INO80,IRAK1,IRAK4,IRF2BP2,IRF3,IRF4,IRF7,IRF8,IRF9,ISG15,ITCH,ITGAM,ITK,ITPKB,JAK1,JAK3,KDM6A,KMT2A,KMT2D,KRAS,LAMTOR2,LAT,LCK,LIG1,LPIN2,LYST,MAD2L2,MAGT1,MALT1,MAP3K14,MAPK8,MBL2,MCM4,MRTFA,MOGS,MPO,MRE11,MS4A1,MSH6,MSN,MTHFD1,MYD88,MYSM1,NBAS,NBN,NCSTN,NEIL3,NFE2L2,NFKB1,NFKB2,NFKBIA,NHEJ1,NHP2,NKX2.5,NLRC4,NLRP1,NLRP12,NLRP2,NLRP3,NOP10,NRAS,NSMCE3,OAS1,ORAI1,OSM,OSTM1,OTULIN,PALB2,PARN,PAX5,PEPD,PGM3,PIGA,PIK3CD,PLEKHG1,PLEKHM1,PMS2,PNP,POLA1,POLD1,POLD2,POLE,POLE2,POLR3A,POLR3C,POLR3F,PRF1,PRKCD,PRKDC,PSEN1,PSENEN,PSMB8,PSMG2,PSTPIP1,PTPRC,PTPRT,RAB27A,RAC2,RAD51,RAD51C,RAG1,RANBP2,RASGRP1,RBCK1,RC3H1,REL,RELA,RELB,RFWD3,RFX5,RFXANK,RFXAP,RHOH,RMRP,RNASEH2A,RNASEH2B,RNASEH2C,RNF168,RNF31,RNU4ATAC,RORC,RPSA,SAMD9,SAMD9L,SAMHD1,SBDS,SEC61A1,SEMA3E,SERPING1,SH3BP2,SH3KBP1,SLC29A3,SLC35C1,SLC39A7,SLC46A1,SLC7A7,SMARCAL1,SMG6,SMIM1,SNX10,SP110,SPINK5,SPPL2A,SRP72,STAT2,STAT4,STIM1,STK4,STN1,STX11,TAP1,TAP2,TAPBP,TAFAZZIN,TBK1,TBX1,TCF3,TCIRG1,TCN2,TERC,TERT,TFRC,TGFBR1,TGFBR2,THBD,TICAM1,TINF2,TIRAP,TLR3,TMC6,TMC8,STING1,TNFAIP3,TNFRSF11A,TNFRSF13B,TNFRSF13C,TNFRSF1A,TNFRSF4,TNFRSF9,TNFSF11,TNFSF12,TOP2B,TP53,TPP1,TPP2,TRAF3,TRAF3IP2,TREX1,TRNT1,TYK2,UBE2T,UNC119,UNC13D,UNC93B1,UNG,USB1,USP18,VPS13B,VPS45,WDR1,WIPF1,WRAP53,XRCC2,ZBTB24,ZNF341
